# Contralateral-Structure-Preserving Endoscopic Resection of Cervical Osteochondroma: A Technical Note

**DOI:** 10.3390/jcm15124575

**Published:** 2026-06-12

**Authors:** Chun-Gon Park, Hyun-Seong Kim, Sung-Kyu Kim

**Affiliations:** 1 Department of Orthopedic Surgery, Chonnam National University Hospital, 42 Jebongro, Dong-gu, Gwangju 61469, Republic of Korea; gonimagic@naver.com; 2 Department of Orthopedic Surgery, St. Carollo Hospital, 221 Sungwang-ro, Suncheon 57931, Republic of Korea; singsing93@hanmail.net; 3 Department of Orthopedic Surgery, Chonnam National University Medical School, 160 Baekseo-ro, Dong-gu, Gwangju 61469, Republic of Korea

**Keywords:** osteochondroma, multiple hereditary exostosis, endoscopy, cervical vertebrae, spine, unilateral biportal endoscopic spine surgery, posterior tension band

## Abstract

**Background:** Cervical osteochondromas invading the vertebral canal are rare but may cause spinal cord compression requiring surgical resection. Conventional open laminectomy may disrupt posterior stabilizing structures and potentially increase the risk of postoperative cervical deformity. This technical note describes a contralateral-structure-preserving endoscopic technique for cervical osteochondroma resection. **Methods:** A 25-year-old man with multiple hereditary exostosis presented with neck pain, mild numbness, and a positive Lhermitte’s sign. Computed tomography and magnetic resonance imaging revealed a 9 × 6 × 10 mm osteochondroma originating from the base of the C3 spinous process and extending into the vertebral canal with spinal cord compression and cord signal change. Preoperative clinical assessment included a Visual Analog Scale (VAS) for neck pain of 6/10, a modified Japanese Orthopedic Association (mJOA) score of 16/18, a Neck Disability Index (NDI) of 30%, and Nurick grade 1. The lesion was treated using unilateral biportal endoscopic spine surgery through a partial unilateral laminectomy and sublaminar endoscopic corridor, aiming for en bloc resection while preserving the contralateral lamina, posterior ligamentous complex, and posterior tension band. Continuous intraoperative neurophysiological monitoring (SSEP and MEP) was used throughout the procedure. **Results:** The osteochondroma was completely resected en bloc using a diamond burr and Kerrison rongeur. Histopathological examination confirmed osteochondroma, and negative margins were identified without residual tumor. The patient’s symptoms resolved completely without postoperative complications, and he was discharged on postoperative day 3. At the 18-month clinical and radiological follow-up, the patient remained symptom-free, with VAS improved to 1–2/10, mJOA improved to 18/18, NDI improved to 4%, and Nurick grade improved to 0, with partial regression of the cord signal change and no evidence of tumor recurrence on follow-up imaging. Cervical lordosis was maintained at the immediate postoperative timepoint. **Conclusions:** Contralateral-structure-preserving endoscopic resection may represent a potential minimally invasive alternative to conventional wide laminectomy or fusion-based approaches in carefully selected cases of benign cervical osteochondroma. Larger comparative studies with long-term follow-up are required to confirm the potential biomechanical and clinical benefits of this approach.

## 1. Introduction

Osteochondromas constitute approximately 10–15% of benign bone tumors [1]. When multiple lesions are present, the condition is classified as multiple hereditary exostosis (MHE), an autosomal dominant disorder caused by mutations in the EXT1 and EXT2 genes [2]. In the spine, osteochondromas are associated with MHE in 95% of cases; approximately 27% of these lesions extend into the vertebral canal and may cause neurologic symptoms [3].

Conventional treatment for osteochondromas invading the vertebral canal typically involves open laminectomy with or without screw fixation [4]. In 2023, Hung et al. reported the endoscopic excision of vertebral canal-invading osteochondromas [5]. However, their technique involved endoscopic en bloc laminectomy, which shares similarities with open procedures in terms of bone resection. In contrast, our technique focuses on a partial unilateral laminectomy that aims to preserve the contralateral lamina and posterior ligamentous complex, thereby potentially minimizing the risk of postoperative kyphosis. To the best of our knowledge, this report introduces a rarely reported contralateral-structure-preserving endoscopic technique for cervical osteochondroma.

## 2. Technical Note

### 2.1. Patient and Imaging Findings

**Figure 1:**
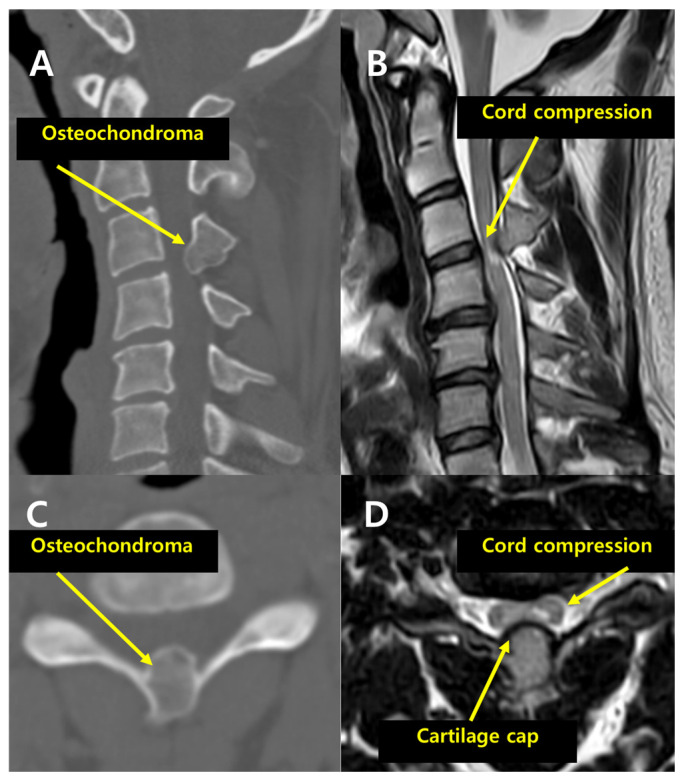
Preoperative imaging of the cervical osteochondroma. (**A**) Sagittal computed tomography showing a bony lesion (arrow) arising from the base of the C3 spinous process. (**B**) Sagittal T2-weighted magnetic resonance imaging demonstrating compression of the cervical spinal cord (arrow) by the lesion, with associated cord signal change. (**C**) Axial computed tomography at the C3 level showing the osteochondroma (arrow) arising from the base of the spinous process and extending into the vertebral canal. (**D**) Axial T2-weighted magnetic resonance imaging at the C3 level confirming compression of the spinal cord (arrow) by the lesion and demonstrating a thin cartilage cap (arrow).

This was a retrospective review of a single patient. The clinical course of the index patient extended from the date of surgical treatment on 15 March 2024 to the final clinical follow-up on 9 September 2025 (18-month clinical follow-up); the last radiological (MRI) follow-up was obtained at 18 months postoperatively. The retrospective review of anonymized medical records and imaging was performed after Institutional Review Board approval (CNUH-EXP-2025-004; 6 January 2025), in accordance with the principles of the Declaration of Helsinki. A 25-year-old man presented with neck pain, mild numbness, and a positive Lhermitte’s sign, which occurred consistently with neck flexion. Motor strength in the upper and lower extremities and gait were normal. Preoperative clinical assessment demonstrated a VAS for neck pain of 6/10, mJOA score of 16/18, NDI of 30%, and Nurick grade 1. The patient had been attending follow-up at our outpatient clinic for MHE since age 17 and had a family history of MHE; all siblings had been diagnosed with the condition. Computed tomography and magnetic resonance imaging revealed a 9 × 6 × 10 mm osteochondroma with a cartilage cap (approximately 1 mm in thickness on T2-weighted MRI) originating from the base of the spinous process of the third cervical vertebra. The lesion extended into the vertebral canal, causing spinal cord compression with associated signal changes in the cord (Figure 1). No preoperative cervical instability was identified.

### 2.2. Surgical Technique

**Figure 2:**
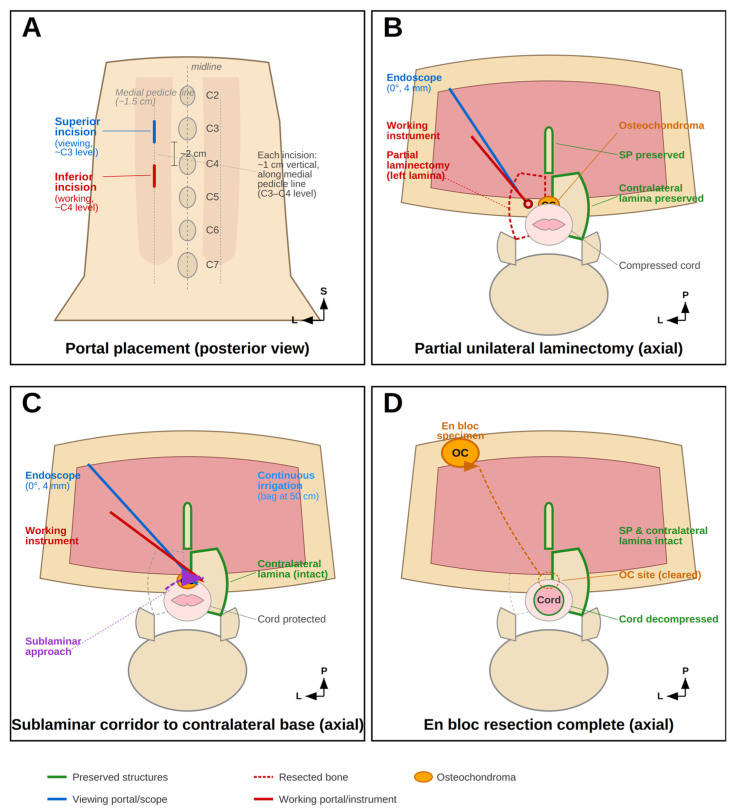
Schematic illustration of the stepwise operative workflow. (**A**) Posterior view showing portal placement: two ~1 cm vertical skin incisions along the medial pedicle line at the C3 (viewing portal) and C4 (working portal) levels on the left side, approximately 1.5 cm lateral to the midline and separated by ~2 cm vertically. (**B**) Axial view of partial unilateral laminectomy of the left C3 lamina (red dashed outline), with the contralateral lamina, spinous process, and posterior ligamentous complex preserved (green). (**C**) Axial view of the sublaminar endoscopic corridor crossing under the spinous process to the contralateral base of the osteochondroma, with continuous saline irrigation maintained at a saline bag height of 50 cm above the patient’s bed level; the grey dashed line indicates the partial unilateral laminectomy (bony window). (**D**) Axial view following en bloc tumor resection, with restoration of cord decompression and complete preservation of the contralateral posterior elements.

Treatment was performed via unilateral biportal endoscopic spine surgery (UBESS). The key technical objective was to achieve complete en bloc resection of the lesion while preserving the contralateral lamina, posterior ligamentous complex, and posterior tension band. The patient was placed in the prone position, with the operating table hinge positioned at the cervical region and the neck moderately flexed (approximately 30°). A spinal endoscopy drape was applied, and the surgeon stood on the left side of the patient. Continuous intraoperative neurophysiological monitoring (SSEP and MEP) was established prior to incision and maintained throughout the procedure. The surgical level was identified using a spinal needle (Becton Dickinson, Franklin Lakes, NJ, USA) and confirmed with C-arm fluoroscopy(Ziehm Vision, Ziehm Imaging, Nuremberg, Germany). Two longitudinal skin incisions, each approximately 1 cm in length, were made along the medial pedicle line at the C3 and C4 levels on the left side (approximately 1.5 cm lateral to the midline), separated by approximately 2 cm vertically; the superior incision served as the viewing portal and the inferior incision as the working portal (Figure 2A). Serial dilators were inserted into each incision toward the lamina of the third cervical vertebra. Triangulation toward the lamina of the third cervical vertebra between the superior viewing port and inferior working portal was confirmed using endoscopy (CONMED Corporation, Largo, FL, USA) and fluoroscopy (Ziehm Vision, Ziehm Imaging, Nuremberg, Germany). A 0° angle, 4 mm diameter endoscope was used throughout the procedure. The saline bag was positioned at 50 cm above the patient’s bed level, and unobstructed saline outflow was meticulously maintained throughout the procedure by careful working sheath positioning and continuous monitoring. This meticulous outflow management is critical for ensuring safe pressure dynamics in cervical endoscopic procedures.

**Figure 3:**
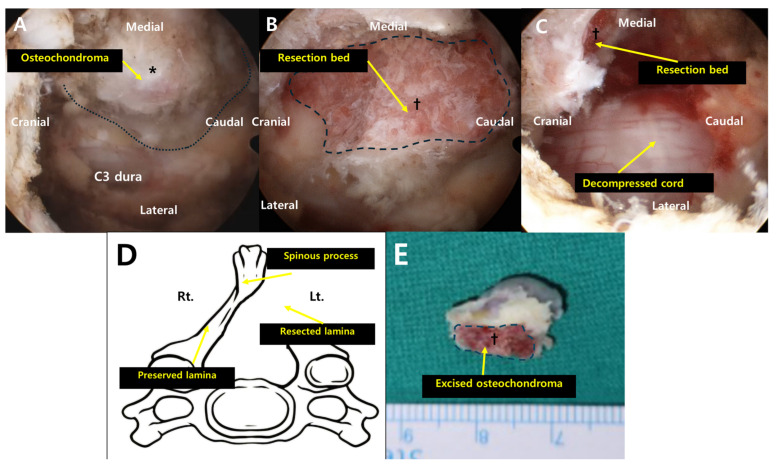
(**A**) Intraoperative view of the osteochondroma (asterisk) and compressed cord (dotted lines). (**B**) Resected base of the spinous process (dagger) and margin (dashed lines). (**C**) Resection bed (dagger) with the decompressed spinal cord exposed. (**D**) Schematic of the sublaminar excision technique. (**E**) En bloc excised osteochondroma (dagger), with the lesion margin outlined by the dashed line and shown against a ruler for scale.

Soft tissues were removed using a radiofrequency ablation system (STARVAC 90°, Smith & Nephew, Andover, MA, USA). Epidural bleeding was controlled primarily using a flowable gelatin-thrombin matrix hemostatic agent (Floseal^®^, Baxter Healthcare, Deerfield, IL, USA), with adjunctive use of radiofrequency ablation at low power settings for focal soft-tissue bleeding and cottonoid pledgets for short-term tamponade when needed. Endoscopic partial left laminectomy of the third cervical vertebra and left flavectomy between the third and fourth cervical vertebrae were then performed using a 4 mm burr (TRAUS SSG10 device, Saeshin Precision Co., Daegu, Republic of Korea) and a Kerrison rongeur (Aesculap, B. Braun, Tuttlingen, Germany). After partial laminectomy and flavectomy, the entire osteochondroma at the base of the spinous process was visualized. Upon confirmation of overall lesion morphology, the cancellous bone of the spinous process above the osteochondroma base was removed using a 2 mm diamond burr (NSK, Nakanishi Inc., Kanuma, Japan). This step was performed with great caution at a low rotational speed of 15,000 RPM with intermittent burr engagement and continuous saline irrigation for cooling, to prevent cervical cord injury from rotational vibration, thermal injury, and dural tearing. Throughout the procedure, the working sheath was carefully positioned to avoid direct compression on the thecal sac. En bloc resection of the osteochondroma was achieved using a burr and a Kerrison rongeur along the right sublaminar area of the third cervical vertebra. A drainage catheter was inserted, and the procedure was successfully completed (Figure 3). The total operative time was 90 min. Estimated blood loss could not be quantified due to continuous saline irrigation; however, no significant intraoperative bleeding was observed. No SSEP or MEP signal changes occurred during the procedure. A schematic illustration of the stepwise operative workflow is provided in Figure 2.

### 2.3. Pathology and Postoperative Outcome

**Figure 4:**
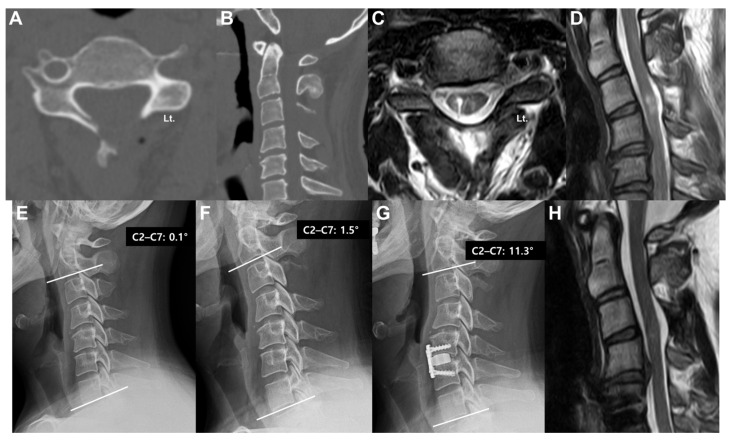
(**A**,**B**) Postoperative axial and sagittal CT. (**C**,**D**) Postoperative axial and sagittal T2-weighted MRI showing resolved cord compression with residual high signal intensity (**D**). (**E**–**G**) Preoperative, immediate postoperative, and final follow-up at 18 months, lateral radiographs (C2–C7 Cobb angles: 0.1°, 1.5°, and 11.3°). The patient underwent C5–C6 anterior cervical diskectomy and fusion (ACDF) for an unrelated disc herniation at 2 months postoperatively (visible in (**G**)). (**H**) Sagittal T2-weighted MRI at 18 months postoperatively, demonstrating partial regression of the cord signal change seen on D and no evidence of recurrence.

Histopathological examination confirmed the diagnosis of osteochondroma. Gross examination demonstrated a C3 spinous process mass measuring 1.3 × 1.2 × 1.0 cm and marginal debris measuring 0.6 × 0.5 × 0.2 cm. Microscopic examination demonstrated histologic features compatible with osteochondroma without cellular atypia, increased mitotic activity, or features suggestive of secondary malignant transformation. Resection margins at the lesion’s base consisted of mature lamellar bone; negative margins were confirmed without evidence of residual tumor. After surgery, the patient’s symptoms resolved without complications, and he was discharged in stable condition on the third postoperative day (Figure 4). At the 18-month clinical follow-up, the patient remained symptom-free. Clinical outcomes were assessed using validated instruments, including the visual analog scale (VAS) for neck pain, the Nurick grade [6], the modified Japanese Orthopedic Association (mJOA) score [7], and the Neck Disability Index (NDI) [8]. Postoperative VAS for neck pain was 1–2/10 (improved from 6/10), mJOA score improved from 16/18 to 18/18, NDI improved from 30% to 4%, and Nurick grade improved from 1 to 0, indicating complete resolution of the preoperative neurological symptoms. Follow-up MRI obtained at 18 months postoperatively demonstrated no evidence of tumor recurrence; postoperative T2 hyperintensity within the cord partially regressed compared with the immediate postoperative MRI. The C2–C7 Cobb angle was 0.1° preoperatively, 1.5° at immediate postoperative imaging, and 11.3° at the final 18-month follow-up. The minimal change at the immediate postoperative timepoint (+1.4°) indicates that the index procedure did not induce acute kyphotic deformity. Two months postoperatively, the patient underwent C5–C6 anterior cervical discectomy and fusion (ACDF) for an unrelated, newly symptomatic disc herniation; this subsequent intervention was not considered a complication of the index procedure.

## 3. Discussion

Endoscopic spine surgery offers a shorter hospital stay and more rapid return to normal activities compared with conventional open surgery due to reduced postoperative pain and bleeding [9]. Conventional open laminectomy for vertebral canal tumors often requires extensive subperiosteal dissection and removal of posterior stabilizing structures, which may lead to postoperative axial pain, iatrogenic cervical kyphosis, and the need for instrumented fixation [10]. In the present technique, the sublaminar endoscopic corridor enabled tumor resection through a limited unilateral bony window while preserving the spinous process, interspinous ligaments, contralateral lamina, and contralateral paraspinal musculature. This limited-access strategy may help maintain posterior tension-band integrity and facilitate early postoperative recovery, although any actual deformity-preventing benefit cannot be established from a single case and warrants confirmation in larger comparative studies.

Hung et al. first reported endoscopic treatment of cervical osteochondroma using an en bloc bilateral laminectomy technique [5]. In contrast, the present technique used a partial unilateral laminectomy to access the sublaminar lesion while leaving the contralateral lamina and posterior ligamentous complex intact. This distinction is clinically relevant because posterior element disruption is a major concern in the cervical spine, particularly when bilateral laminectomy is performed. Schmeiser et al. compared bilateral and unilateral laminectomy approaches and reported that bilateral approaches required fusion in 92% of cases to prevent postoperative kyphosis, whereas unilateral approaches achieved comparable clinical outcomes with fusion rates of 67% while better preserving contralateral structures and cervical lordosis [11].

Patient selection is critical for the safe application of this technique. Suitable indications include the following: (1) benign cervical osteochondroma with a sublaminar or paramedian location amenable to a unilateral corridor; (2) cartilage cap less than 2 cm without imaging features of malignant transformation; (3) small to moderate lesion size permitting en bloc resection through a partial unilateral laminectomy; and (4) absence of preoperative cervical instability. Contraindications include the following: (1) suspected malignant transformation (cartilage cap greater than 2 cm, irregular borders, rapid growth, or soft-tissue extension); (2) heavily calcified or giant lesions requiring wide marginal resection; (3) lesions involving the vertebral artery foramen or extending into the anterior column; (4) preoperative segmental instability mandating instrumented fusion; and (5) multi-level pathology requiring bilateral exposure [1,3,4,12].

A range of surgical options exists for cervical-canal-invading osteochondromas, each with distinct advantages and limitations. Open bilateral laminectomy provides wide exposure but disrupts the posterior tension band and frequently requires instrumented fusion to prevent postoperative deformity [10,11,13]. Laminoplasty preserves posterior structures via a hinge technique but is generally indicated for multi-level decompression rather than focal tumor resection. Tubular microscopic decompression and uniportal full-endoscopic techniques offer reduced soft-tissue dissection but provide a more limited working space than the biportal approach. In particular, lesions requiring bimanual maneuvering—such as en bloc tumor resection—are more readily handled in the biportal configuration, in which the endoscope and instruments occupy separate portals [14,15,16,17]. The bilateral biportal endoscopic technique described by Hung et al. preserves muscle but still entails bilateral bony resection [5]. The present technique seeks to combine the advantages of these approaches: it minimizes soft-tissue dissection, preserves the contralateral lamina and posterior ligamentous complex, and avoids fusion, while providing the bimanual working space inherent to biportal endoscopy.

Cervical myelopathy is commonly graded as mild, moderate, or severe based on the mJOA score, and our patient had a relatively mild preoperative clinical presentation, with an mJOA score of 16/18 [7]. The mJOA alone, however, may not reliably discriminate progressive clinical changes, particularly in severe forms, as Martin et al. reported substantial inter-observer variability despite good overall reliability [18]. A comprehensive assessment by the treating surgical team, therefore, remains essential to ensure holistic decision-making. In this case, postoperative VAS improved from 6 to 1–2, mJOA score improved from 16/18 to 18/18, NDI improved from 30% to 4%, Nurick grade improved from 1 to 0, and cervical lordosis was maintained at the immediate postoperative timepoint. T2 hyperintensity within the cord partially regressed but did not fully resolve on the 18-month follow-up MRI, despite complete clinical recovery. As previously demonstrated by Vedantam et al., residual T2 hyperintensity following cervical decompression may persist for many months or years and is generally believed to reflect chronic gliotic or myelomalacic changes rather than ongoing compression [19]. The incomplete resolution of the T2 signal change despite full clinical recovery in our case is consistent with this established radiologic–clinical dissociation.

Most previously reported cervical osteochondromas have been treated using conventional open laminectomy, often involving bilateral exposure and partial disruption of posterior stabilizing structures. Reports of minimally invasive or endoscopic approaches remain limited, and cases demonstrating complete contralateral posterior element preservation are exceedingly rare. In this context, the present technical note expands the limited literature by demonstrating that selected benign cervical canal lesions may be resected endoscopically without routine bilateral laminectomy or fusion.

However, this technique has limitations. It has a steep learning curve and may not be suitable when a wide marginal excision is required, such as in cases of malignant tumors or giant lesions. In our patient, endoscopic resection was feasible because the lesion was a benign osteochondroma. Malignant transformation should be suspected based on a constellation of features—including cartilage cap thickness greater than 2 cm on MRI, rapid growth, irregular borders, soft-tissue extension, and clinical findings—rather than cartilage cap thickness alone [12,20]. Since the cartilage cap in this case measured only approximately 1 mm on MRI and the lesion showed no concerning radiologic features, marginal excision via endoscopy was appropriate and achievable.

The application of UBESS to the cervical spine requires particular attention to safety and neurological preservation. Recent evidence supports the safety and efficacy of UBESS for selected cervical posterior procedures. Lee et al. conducted a meta-analysis of 390 patients undergoing unilateral biportal endoscopic posterior cervical foraminotomy and reported an overall complication rate of 6.2%, with dural tear as the most common complication at 2.1% and transient neurological deficits at 0.8% [21]. In a prospective randomized controlled trial, Peng et al. directly compared UBESS to anterior cervical decompression and fusion and demonstrated equivalent clinical pain relief and functional improvement, with overall complications of 14.3% in the UBESS group versus 25% in the fusion group, and no C5 nerve root palsy in the UBESS cohort compared with 5.9% in the fusion group [22]. Although these studies were not conducted in tumor populations, they support the technical feasibility and safety profile of UBESS in selected cervical posterior procedures, providing indirect support for its cautious application to carefully selected benign tumor-related pathology.

Several procedural risks warrant explicit discussion. The most demanding step is contralateral sublaminar drilling, which carries a theoretical risk of cord injury; in our practice, this risk was mitigated by low-RPM drilling (15,000 RPM) with intermittent burr engagement, careful working sheath positioning to avoid direct thecal sac compression, and continuous SSEP and MEP monitoring. Epidural bleeding was managed primarily using a flowable gelatin-thrombin matrix hemostatic agent (Floseal^®^, Baxter Healthcare, Deerfield, IL, USA), with adjunctive radiofrequency ablation for focal soft-tissue bleeding. Saline irrigation pressure was managed by maintaining the saline bag 50 cm above the patient’s bed level and continuously monitoring outflow, in line with established cervical UBESS safety protocols [23,24]. No dural tear occurred in our case; however, small tears can typically be managed endoscopically with fibrin sealant, while larger tears may require conversion to open or microscopic repair.

Endoscopic tumor resection for spinal pathology has been increasingly reported in recent years; however, endoscopic excision of cervical osteochondromas causing spinal cord compression remains exceptionally rare. To the best of our knowledge, only one prior case has described posterior endoscopic resection of a cervical osteochondroma invading the vertebral canal and causing myelopathy [5]. The present case, therefore, represents a rare additional report of posterior endoscopic resection for vertebral-canal-invading cervical osteochondroma and, to the best of our knowledge, appears to be the first to describe a partial unilateral laminectomy technique with complete preservation of the contralateral posterior elements. This technical distinction may be particularly relevant in the cervical spine, where preservation of posterior stabilizing structures has been associated with improved alignment outcomes in comparative studies of laminectomy approaches [11].

This study has several inherent limitations. First, the report describes a single case, and the generalizability of the findings is therefore limited. Second, the retrospective design precluded prospective standardized outcome assessment; in particular, postoperative dynamic flexion-extension radiographs were not obtained. Third, the patient underwent C5–C6 ACDF two months postoperatively for an unrelated, newly symptomatic disk herniation. Although this intervention was not a complication of the index procedure, it may have confounded the long-term clinical outcome assessment. Specifically, postoperative VAS for neck pain, mJOA score, NDI, and Nurick grade measured beyond two months cannot be attributed exclusively to the index endoscopic resection, as the ACDF likely contributed to both pain relief and functional improvement. Similarly, the long-term cervical alignment outcome was confounded by the ACDF, so the immediate postoperative Cobb angle measurement should be considered the primary alignment outcome attributable to the index procedure, and the long-term biomechanical benefit of contralateral structure preservation cannot be confirmed from this single case. Fourth, the technique requires substantial prior endoscopic experience and a steep learning curve, which may limit reproducibility. Fifth, an intraoperative video and a representative pathology slide image were not available. A larger comparative series with standardized clinical outcome measures and longer follow-up is required to confirm the potential biomechanical and clinical benefits of this approach.

## 4. Conclusions

This technical note demonstrates the feasibility of contralateral-structure-preserving endoscopic resection for a selected case of cervical osteochondroma invading the vertebral canal. Using a partial unilateral laminectomy and sublaminar endoscopic corridor, complete en bloc tumor resection was achieved while preserving the contralateral lamina, posterior ligamentous complex, and posterior tension band. This technique may represent a potential minimally invasive alternative to conventional wide laminectomy or fusion-based approaches in carefully selected benign cervical osteochondromas; however, further cases and comparative studies with long-term follow-up are required to confirm its safety, reproducibility, and potential biomechanical benefits.

## Data Availability

The data are not publicly available due to patient privacy concerns but are available from the corresponding author on reasonable request.
